# Effect of Steel Fiber on the Strength and Flexural Characteristics of Coconut Shell Concrete Partially Blended with Fly Ash

**DOI:** 10.3390/ma15124272

**Published:** 2022-06-16

**Authors:** Ramaiah Prakash, Nagarajan Divyah, Sundaresan Srividhya, Siva Avudaiappan, Mugahed Amran, Sudharshan Naidu Raman, Pablo Guindos, Nikolai Ivanovich Vatin, Roman Fediuk

**Affiliations:** 1 Department of Civil Engineering, Alagappa Chettiar Government College of Engineering and Technology, Karaikudi 630001, India; 2 Department of Civil Engineering, Government College of Technology, Coimbatore 641013, India; divyah991@gmail.com; 3 Department of Civil Engineering, Varuvan Vadivelan Institute of Technology, Dharmapuri 636703, India; srividhya132@gmail.com; 4 Departamento de Ingeniería Civil, Universidad de Concepción, Concepción 4070386, Chile; savudaiappan@udec.cl; 5 Centro Nacional de Excelenciapara la Industria de la Madera (CENAMAD), Pontificia Universidad Católica de Chile, Av. Vicuña Mackenna 4860, Santiago 8330024, Chile; pguindos@uc.cl; 6 Department of Civil Engineering, College of Engineering, Prince Sattam Bin Abdulaziz University, Alkharj 16273, Saudi Arabia; 7 Department of Civil Engineering, Faculty of Engineering and IT, Amran University, Amran 9677, Yemen; 8 Civil Engineering Discipline, School of Engineering, Monash University Malaysia, Jalan Lagoon Selatan, Bandar Sunway 47500, Selangor, Malaysia; sudharshan.raman@monash.edu; 9 Peter the Great St. Petersburg Polytechnic University, 195251 St. Petersburg, Russia; vatin@mail.ru (N.I.V.); fedyuk.rs@dvfu.ru (R.F.); 10 Polytechnic Institute, Far Eastern Federal University, 690922 Vladivostok, Russia

**Keywords:** coconut shell, fly ash, steel fiber, flexural strength, ductility

## Abstract

The construction industry relies heavily on concrete as a building material. The coarse aggregate makes up a substantial portion of the volume of concrete. However, the continued exploitation of granite rock for coarse aggregate results in an increase in the future generations’ demand for natural resources. In this investigation, coconut shell was used in the place of conventional aggregate to produce coconut shell lightweight concrete. Class F fly ash was used as a partial substitute for cement to reduce the high cement content of lightweight concrete. The impact of steel fiber addition on the compressive strength and flexural features of sustainable concrete was investigated. A 10% weight replacement of class F fly ash was used in the place of cement. Steel fiber was added at 0.25, 0.5, 0.75, and 1.0% of the concrete volume. The results revealed that the addition of steel fibers enhanced the compressive strength by up to 39%. The addition of steel fiber to reinforced coconut shell concrete beams increased the ultimate moment capacity by 5–14%. Flexural toughness was increased by up to 45%. The span/deflection ratio of all fiber-reinforced coconut shell concrete beams met the IS456 and BS 8110 requirements. Branson’s and the finite element models developed in this study agreed well with the experimental results. As a result, coconut shell concrete with steel fiber could be considered as a viable and environmentally-friendly construction material.

## 1. Introduction

In the building sector, concrete is the most extensively used construction material. After water, it is the second most-consumed material by humans [1]. The manufacture of concrete significantly depletes natural resources [2,3]. There is a legal need to employ alternative and renewable resources in concrete production to maintain the ecological balance [4]. It can be observed that concrete ingredients such as cement and aggregates can be substituted from the industrial, household, and agricultural sectors [4,5,6,7,8,9,10,11,12]. Aggregates make up around 70–80% of the overall volume of concrete [13]. The most significant disadvantage of employing natural aggregate in concrete is the issue of sustainability [14]. As a result, the concrete sector must seek out other, long-term building materials [15,16,17,18,19]. Using waste materials, industrial by-products, and recycled aggregate as an alternative to concrete ingredients is one of the most acceptable ways to achieve sustainability in concrete production [20,21,22,23,24,25].

**Figure 1:**
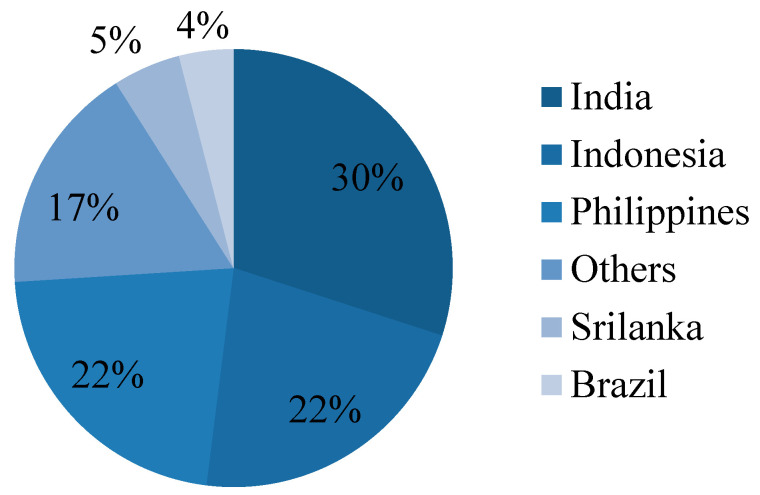
The production of coconut nuts worldwide.

**Figure 2:**
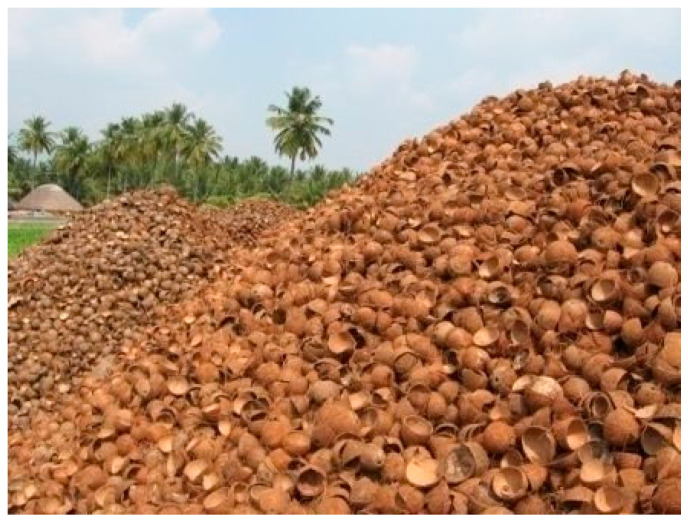
The discarded coconut shells.

Coconut shell aggregate from agricultural waste has lately been used in concrete manufacturing [26,27,28]. Coconut farms can be found in a variety of nations including India. According to the 2015 report of the Asian and Pacific Coconut Community, 67 billion coconut nuts are produced worldwide. Figure 1 shows the production of coconut worldwide as per the statistics of the Coconut Development Board. Over 23,904 million coconut nuts were collected in 2017–2018, according to India’s Ministry of Agriculture and Farmers’ Welfare, which accounts for almost 30% of global coconut production [29]. This large-scale coconut harvesting also contributes to an increase in agricultural waste. Figure 2 shows the dumped discarded coconut shell waste in the copra processing field. When the discarded coconut shell is used in concrete, it has various advantages including lowering the cost of concrete production and reducing granite rock depletion. The utilization of coconut shell concrete in house construction will contribute to low-income families in terms of economy [30]. The adoption of coconut shell concrete decreases the dependence on natural granite aggregate. Furthermore, the disposal and recycling problems of coconut shell waste have become much more accessible. Thus, it may be a novel approach and considered as a sustainable eco-friendly concrete ingredient. According to Olanipekun et al. [31], using coconut shells in concrete can save 30% on the construction costs, so the usage of coconut shell concrete in house construction will benefit low-income families in cost savings [30]. As a result, coconut shell can be used in concrete to reduce the reliance on natural granite aggregate.

Furthermore, the challenges of the disposal and recycling of coconut shell trash have been greatly simplified. As a result, it might be an innovative method and a sustainable approach to concrete production for the benefit of the construction industry. Basri et al. [32] claimed that once wood-based components are embedded in concrete, they will not contaminate or leach harmful compounds. The bonding behavior of coconut shell aggregate with the concrete matrix is compatible, and it does not require any preparation before its use in concrete. It also has a low inhibitory effect. In terms of durability, coconut shell concrete fared similarly to other lightweight concretes [28,30]. Coconut shell concrete is more impact resistant than normal concrete [26]. Because of the lightweight characteristic of coconut shell aggregate, concrete made with it has a density within 2000 kg/m^3^, putting it in the lightweight concrete category. Reduced dead load, less seismic stresses, simpler formwork, reduced foundation area, enhanced fire resistance, better thermal insulating characteristics, good sound absorption behavior, better frost resistance, enhanced hydration of concrete, and easy transport of concrete are just a few of the benefits of lightweight aggregate concrete (LWAC). It also has a lower stiffness, which results in fewer micro-cracks, and a uniform distribution of cracks at the micro-level, which increases its durability in harsh environments [33].

Due to its enormous CO_2_ emissions, the production of cement is a significant contributor to global warming [34]. Concrete production, as an eco-friendly element, is difficult due to the usage of cement [35,36]. Cement is consumed in the range of 200 to 1200 kg per cubic meter of concrete. Cement manufacturing industries account for 5% of the total global CO_2_ emissions. Furthermore, according to the research in this field, cement manufacturing accounts for 85% of the total CO_2_ emissions during the lifecycle of concrete structures [37,38,39]. It has been used as a locally generated by-product as supplementary cementitious materials and other low-carbon resources in the manufacturing of concrete in order to reduce the reliance on cement in the production of concrete [40,41,42]. In this study, fly ash, created during coal combustion in thermal power plants, was employed as a partial substitute for cement. Fly ash’s environmental impact and disposal have caused problems for power plants. The usage of fly ash solves the problem of disposal and reduces the amount of greenhouse gases released into the environment [43,44,45,46,47,48]. The use of fly ash to replace a significant amount of cement in the concrete and concrete producing industry can significantly cut carbon emissions. Fly ash has a promising performance in concrete due to its physical, chemical, and mineralogical qualities. Furthermore, the spherical form of fly ash lowers the concrete’s water–cement ratio.

Because lightweight concrete (LWC) is 20–25% lighter than conventional concrete, its cheaper handling and shipping costs make it an attractive material [49]. LWC has a high degree of flexibility, a low dead weight, excellent seismic resistance, and minimal cost of foundation [50]. The use of precast LWC parts results in more inadequate transportation and fixing expenses [51]. The most significant drawback of LWC is that it needs a high amount of cement to reach a similar strength to that of traditional concrete. However, this disadvantage could be mitigated by replacing pozzolanic industrial waste for a portion of the cement. Karmegam et al. [52] discovered that the use 10% fly ash in the place of cement increased the strength of oil palm shell (OPS) LWC. Similarly, Prakash et al. [53] found that replacing fly ash at the rate of 10% in coconut shell concrete increased the strength of the concrete considerably.

Incorporating fibers into construction materials, on the other hand, has been practiced in various regions worldwide since ancient times. The necessity to improve the tensile capacity of the materials’ ‘perceived’ brittle properties was the driving force behind this work. This method was used to create fiber reinforced concrete (FRC) in the 20th century, which gained popularity in the building sector because of its increased strength. Steel fiber incorporation in the cement matrix improves the toughness, yield load, first cracking load, moment of resistance, and crack resistance of reinforced concrete beams subjected to transverse loading, according to research on fiber-reinforced concrete [54,55,56,57,58,59,60]. The improvement in the ductility for fiber addition, on the other hand, is a critical characteristic for structural concrete members. Steel fiber addition to concrete has yielded mixed results in terms of increasing or decreasing the ductility in FRC beams [42,61,62,63,64,65]. The addition of fibers to improve the structural behavior such as shear, impact resistance, service load behavior, crack management, and so on might reduce the ductility under flexural loading [63]. Steel fiber reinforced coconut shell concrete beams are comparable to coconut shell concrete beams in terms of flexural performance.

Although there are plenty of previous studies on the mechanical characteristics of fiber-reinforced coconut shell concrete, the research into the flexural characteristics of steel fiber-reinforced coconut shell concrete RC beams through analytical and numerical analysis is rare. Hence, this study explored the influence of steel fibers at 0.25%, 0.5%, 0.75%, and 1.0% with a constant fly ash replacement of 10% on the compressive strength and flexural behavior of coconut shell concrete beams. Additionally, this study investigated the impact of steel fiber addition on the load-carrying capacity, crack resistance, and ductility of the steel fiber-reinforced coconut shell reinforced concrete beams under flexural testing to analyze its feasibility for use as structural concrete. The analytical and numerical analysis was carried out and the results were compared with the experimental results.

## 2. Materials and Methods

### 2.1. Materials

**Figure 3:**
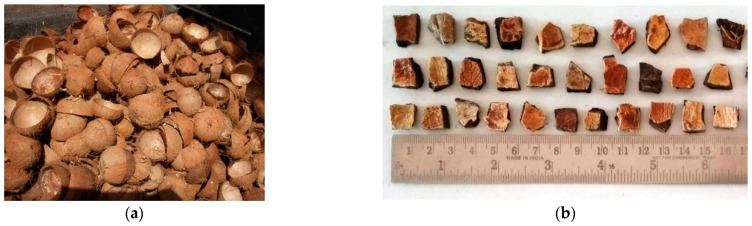
(**a**) Coconut shell and (**b**) coconut shell aggregate.

The binder taken for this investigation was ordinary Portland cement (OPC, Asthivaram, Burma Colony, Karaikudi SIDCO Industrial Estate, Sivaganga, Tamil Nadu, India) grade 53 with a 3350 cm^2^/g specific surface area and 3.13 specific gravity as specified by IS:12269-1987 [66]. As a supplementary cementitious material, class F fly ash was used, which has a 7290 cm^2^/g specific surface area and 2130 kg/m^3^ density, respectively. As a fine aggregate, river sand confirmed as Zone II was employed. The coconut shell was taken from a local copra processing yard. The coconut shell was then crushed into smaller pieces. Crushed coconut shells with sizes of 12.5–4.75 mm were utilized as the coarse aggregate. Clean water was used to wash the produced coconut shell aggregate, which was then dried in the sun. Coconut shell aggregates require immersion in clean water for 24 h due to their high rate of moisture retention capacity. To attain the saturated surface dry (SSD) state of the coconut shell concrete, the soaked aggregates were then air-dried. The SSD state aggregate will not absorb water further, ensuring that the workability of the final product is unaffected. Figure 3a depicts the copra processing yard’s accumulated coconut shell waste. Figure 3b depicts the crushed coconut shell aggregates. The physical characteristics of the coconut shell aggregate were found experimentally and are listed in Table 1. Conplast SP430 superplasticizer (RMS Associates, Dindigul, Tamil Nadu, India) was utilized, and bore well water from inside the college campus was used for concrete mixing. Hooked-end steel fibers (length = 35 mm, dia. = 0.75 mm) were utilized. The fibers utilized had a tensile strength of 1100 MPa.

### 2.2. Mixing Proportion

In total, five mixes for the plain coconut shell and steel fiber-reinforced coconut shell concrete beams were cast. Table 2 shows the various mix proportions for the fiber-reinforced coconut shell concrete. All ingredients were kept the same in all mixes as that of the control mix except for the addition of steel fiber contents of 0.25%, 0.50%, 0.75%, and 1.00%.

### 2.3. Testing of Specimens

**Figure 4:**
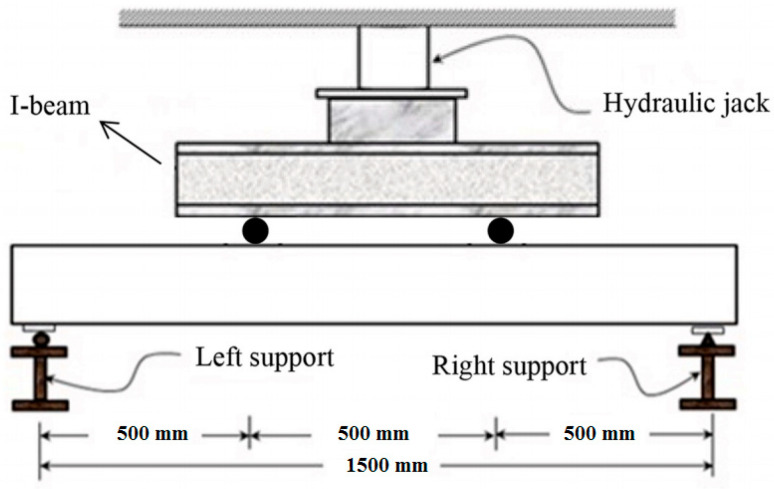
The flexural strength experimental setup.

**Figure 5:**
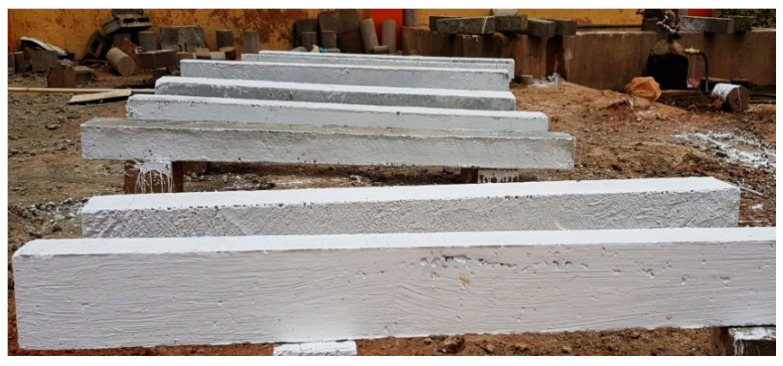
The RC beams cast for flexural testing.

The compressive strength test on 100 mm cube specimens was tested as per IS:516-2014. The flexural strength was determined using 100 mm × 150 mm × 1700 mm size reinforced concrete beams. The bottom side of the RCC beams was reinforced with two 10 mm diameter bars. The top side was reinforced with two 8 mm diameter bars. Stirrups of 6mm diameter bars at 90 mm center to center were provided at the supports. The flexural strength test was carried out in a 500 kN loading frame. The experimental setup consisted of four-point loading with pinned supports on both ends of the beam. The pinned supports at a span of 1500 mm were used. The distance between the point loads was 500 mm. The load was applied at a loading rate of 2.3 kN/s until ultimate failure using a load cell connected to the datalogger. The linear variable displacement transducers (LVDT, Metal LVDT Transducers, Bengaluru, India) were used to determine the deflection at the midspan and one-fourth point of span. The deflection was measured for each load increment. The experimental setup is shown in Figure 4. The beams cast to test its flexural performance are shown in Figure 5.

## 3. Result and Discussion

### 3.1. Compressive Strength

**Figure 6:**
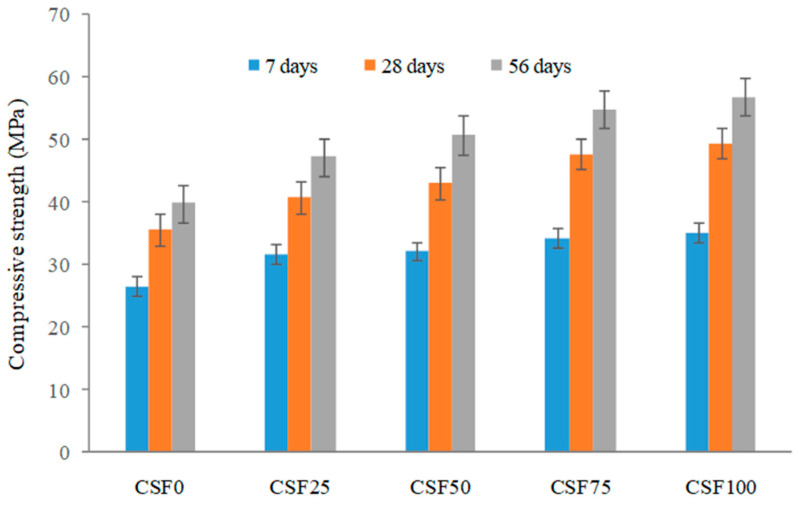
The compressive strength of coconut shell concrete reinforced with steel fibers.

As is widely-known, steel fiber addition has a stronger influence on the concrete compressive strength than synthetic fibers. Steel fibers have a higher strength, length, and modulus of elasticity than the other fibers. Figure 6 depicts the compressive strength of steel fiber-reinforced concrete mixtures used in this investigation. The compressive strength of the mixes increased as the steel fiber % increased. The crack-bridging action of steel fibers boosted the compressive strength of LWC. As the compressive stress grew, cracks appeared and progressed through the concrete’s weak plane. The coconut shell concrete’s weak plane was caused by a lack of CS–cement mortar bonding. When the crack reached a randomly dispersed steel fiber, the fiber bridging caused the crack to close, carrying the crack edge stress between the fiber–mortar interface bonds and boosting compressive strength. The steel fiber addition to LWC greatly improved the compressive strength [67]. The steel fiber with expanded clay aggregate improved the compressive LWC, according to a previous study [68]. Wang and Wang [69] increased the compressive strength of LWC by 23% by using lightweight shale aggregate. Lee and Song [70] found that adding 1% steel fiber to LWC utilizing cellular expanded shale aggregate increased the compressive strength by 37%. In this investigation, steel fiber added to coconut shell concrete enhanced the compressive strength from 15% to 39%. The highest compressive strength of 49.5 MPa was discovered with a 1% fiber addition. The increase in compressive strength at a high steel fiber volume was attributed to some degree of restraint provided by the steel fiber, which was believed to induce the confinement effect in concrete. Due to the confinement effect, the cube’s lateral expansion was minimized, resulting in an increase in the load carrying capacity.

### 3.2. Flexural Behavior

#### 3.2.1. Mode of Failure

**Figure 7:**
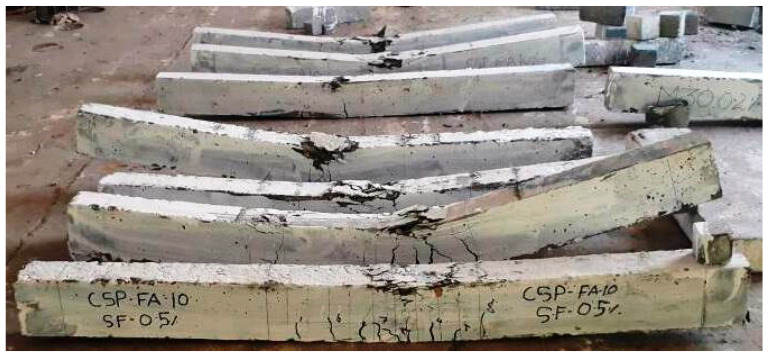
The beams tested for flexure (CS, CSF25, CSF 50, CSF 75, and CSF100).

**Figure 8:**
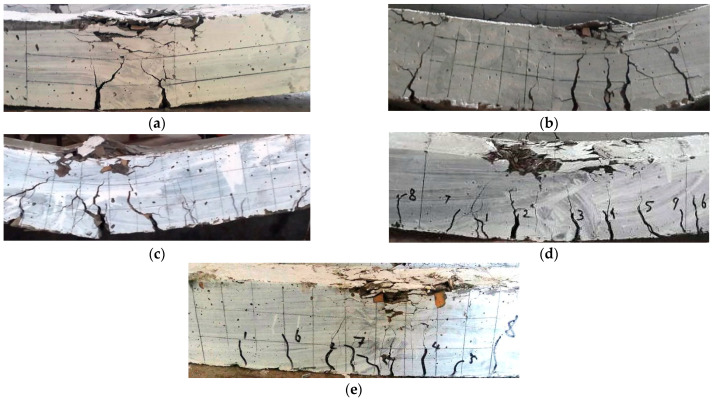
The modes of failure of the CS beams: (**a**) CS, (**b**) CSF25, (**c**) CSF 50, (**d**) CSF 75, and (**e**) CSF100. Annotations: The numbers 1–9 indicate the cracks appearing on the concrete surface in sequence.

All of the specimens (Figure 7) showed typical flexural failure since they were planned and produced as under-reinforced sections. Figure 8a–e depicts the failure mechanisms of all of the beams evaluated in the fiber-reinforced coconut shell concrete mixtures. In the constant moment zone, the number of vertical parallel flexural fractures was produced until the longitudinal steel bar yielded. When the load reached its maximum, the cover of the beam in the compression zone began to break and the crushing of concrete occurred in the zone of compression. Crushing of the concrete in the compression zone caused the coconut shell concrete beam to collapse, as illustrated in Figure 8a. The presence of steel fibers to the coconut shell concrete beams, on the other hand, altered the failure pattern of the beam. Figure 8b–e depicts that all of the steel fiber reinforced beams failed due to the longitudinal main steel bar fracture in the constant moment zone, resulting in concrete wedge formation (concrete crushing).

#### 3.2.2. Moment Capacity

The experimental moment capacity values beams are shown in Table 3. The ultimate moment of the non-fibrous coconut shell concrete beams was 8.1 kNm. The inclusion of the steel fiber enhanced the ultimate moment capacity by 5–14%, as expected. The ultimate moment capacity of beams is increased as the fiber content increases. The highest ultimate moment capacity of the beam specimen was recorded as 9.3 kNm for the specimen CSF100 beam with 1% steel fiber addition, which was about 14% (or 1.2 kNm) greater than the ultimate moment of the non-fibrous coconut shell concrete beam. The crack bridging effect improved the mechanical characteristics of the steel fiber-reinforced coconut shell concrete samples. Steel fibers improved the mechanical characteristics of coconut shell concrete via the interfacial adhesion of fibers to the matrix. The fiber matrix interfacial bond permits the fiber to take up the fracture tip stress partially or entirely as the crack advances the fiber by bridging the cracks across in the matrix. Extra energy is needed to overcome the interfacial bond, thus crack progression and opening are slowed, crippled, or even prevented, therefore enhancing the energy capacity of the coconut shell concrete beam.

Furthermore, when the fraction of the steel fiber addition was less than 0.50%, the beam generated a small increase in the moment capacity. The increase is expected to be between 8% and 12%. In comparison to a plain coconut shell concrete beam, when the steel fiber addition exceeded 0.5% volume fraction (0.75 and 1%), the beam generated a significant improvement in moment capacity (10–14%). This result indicates that a minimum of 0.5% steel fiber is required to significantly increase the moment capacity of steel fiber-reinforced coconut shell concrete. Because the fibers are randomly dispersed across the cross section of steel fiber-reinforced coconut shell concrete beams (tension and compression zones), the tension and compression toughness is enhanced, resulting in a significant increase in the flexural load capacity.

The actual and theoretical moments of a steel fiber reinforced coconut shell concrete beam are compared in Table 3. The code IS 456-2000 was used to compute the theoretical moment capability. The moment capacity of the steel fiber-reinforced coconut shell concrete beams was undervalued because the fibers were not taken into consideration when the moment capacity was calculated using the code of practice. To integrate the effects of fibers on the moment capacity of the steel fiber-reinforced concrete beams and create an empirical formula to estimate the moment–deflection relationship of steel fiber reinforced concrete beams, more research is needed on the combined effect of reinforcement and fiber.

#### 3.2.3. Deflection Characteristics

**Figure 9:**
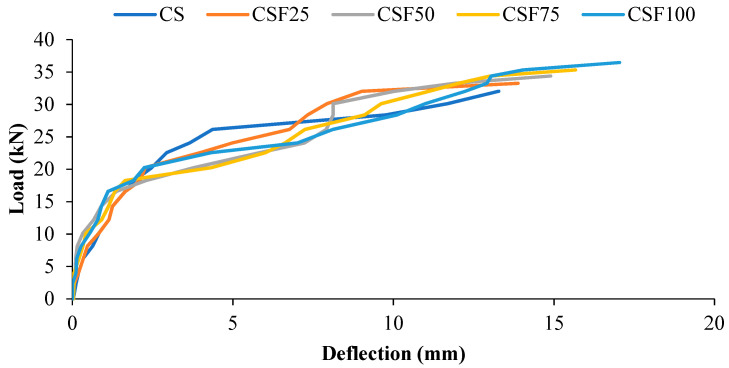
The load–deflection curve of the fiber reinforced-coconut shell concrete mixes.

The load–deflection behavior of all specimens with and without steel fibers is shown in Figure 9. All of the beams had a similar load–deflection curve pattern. Before a fracture occurs in any beam, the slope of the moment–deflection curve is observed to be steep and linear. Steel reinforcement and concrete area both contribute to the beam’s rigidity. Once the flexural fractures occurred and the stiffness was reduced, the slope of the moment–deflection plot changed, but the slope remained linear until the longitudinal steel bar yielded. The load–deflection plot revealed a substantial enhancement of deflection with little rise in load at the latter stages.

Table 4 shows the deflections of all of the coconut shell concrete beams (with and without fibers) under various loads. At ultimate load, the beam (without fiber) exhibited a deflection of approximately 13.28 mm. In the coconut shell concrete beam, the inclusion of steel fiber increased the deflection at the final stage by about 5 to 28%. The deflection of the fiber-reinforced beams at ultimate load ranged from 13.9 to 17.0 mm compared to 13.28 mm for the plain beams. Furthermore, the deflection of the beams rises as the volume % age of the steel fibers increases. In the beam reinforced with 1% steel fiber, a maximum deflection of 17.0 mm was measured. The inclusion of fiber enhanced the ductility of the RC beams, resulting in an increase in the ultimate deflection. A similar conclusion may be drawn at the stage of service load as per IS 456-2000.

The ratio between the span and service load deflection should not exceed 250 in order to fulfil the serviceability limit state as defined by IS456 and BS 8110. The aggregate stiffness was influenced by the porous structure and low density of CS. As a result, the modulus of elasticity of concrete produced using the CS aggregate was low. Due to the low modulus of elasticity of the coconut shell concrete, the displacement within the service loads for singly reinforced beams is acceptable since the span–deflection ratios of the mix CS were 555, which is well within the permitted limit set by IS456:2000 and BS 8110. To meet the serviceability standards of the beams, IS 456 and BS 8110 propose a permitted limit of span/250 for the deflection. According to IS456 and BS 8110, the span/deflection ratio of the fiber-reinforced coconut shell concrete such as CSF25, CSF50, CSF75, and CSF100 is likewise within the permitted range.

Once the yield stage is reached, the fibers play a critical role in the post-yielding flexural characteristics of the steel fiber-reinforced beams. The difference in deflection at the ultimate and yield stages of the beam can be used to achieve this. Once the bars yielded, the applied load was not only accepted by the reinforcement, but was also transferred from the steel reinforcement to the concrete in order to attain a new equilibrium. The steel fiber led to fiber–matrix interfacial bonding, which enhanced the concrete beam’s load bearing capability over a protracted deflection. In the yielding and final phases, this improves the toughness of the steel fiber-reinforced coconut shell concrete beams.

#### 3.2.4. Analytical Model for Deflection of Coconut Shell Concrete Beams

Branson [71] suggested Equation (1) as a formula for the effective moment of inertia of a beam.

(1) $$I_{e}=I_{g}{\left(\frac{M_{cr}}{M_{a}}\right)}^{m}+I_{cr}\left\{1-{\left(\frac{M_{cr}}{M_{a}}\right)}^{m}\right\}$$

where *I_g_* represents the gross moment of inertia without reinforcement, while *I_cr_* represents the cracking moment of inertia. The service moment is *M_a_*, and the first cracking moment is *M_cr_*. Deflection at mid-span for each beam with two symmetrical points load is calculated using Equation (2).

(2) $$\Delta =\frac{M_{a}\left(3L^{2}-4a^{2}\right)}{24EI}$$

where *M_a_* is the moment acting on the beam; “*a*” is the distance between the load location and the support; *E* is the concrete’s elastic modulus; *L* is the beam’s span; and *I* is the moment of inertia. Because *I* is not constant while the concrete beam is loaded, it is substituted by the term *I_e_*, which gives the effective value of the moment of inertia of the beam. Deflection can be precisely determined experimentally. When the experimental value is entered into Equation (3), the experimental *I_e_* may be calculated as shown in Equation (3).

(3) $$I_{e(\exp)}=\frac{M_{a}\left(3L^{2}-4a^{2}\right)}{24E\Delta_{exp}}$$

From Equation (1), the power value (*m*) can be written as:

(4) $$m=\frac{log\frac{\left(I_{e}-I_{cr}\right)}{\left(I_{g}-I_{cr}\right)}}{log\left(\frac{M_{cr}}{M_{a}}\right)}$$

Regression analysis for the whole service load was conducted using the experimental moment of inertia obtained. The results suggested that the average power value (*m*) in Branson’s calculation for beams should be about 4.2. The revised Branson’s equation for coconut shell concrete beam is given by Equation (5).

(5) $$I_{e}=I_{g}{\left(\frac{M_{cr}}{M_{a}}\right)}^{4.2}+\left\{1-{\left(\frac{M_{cr}}{M_{a}}\right)}^{4.2}\right\}$$

**Figure 10:**
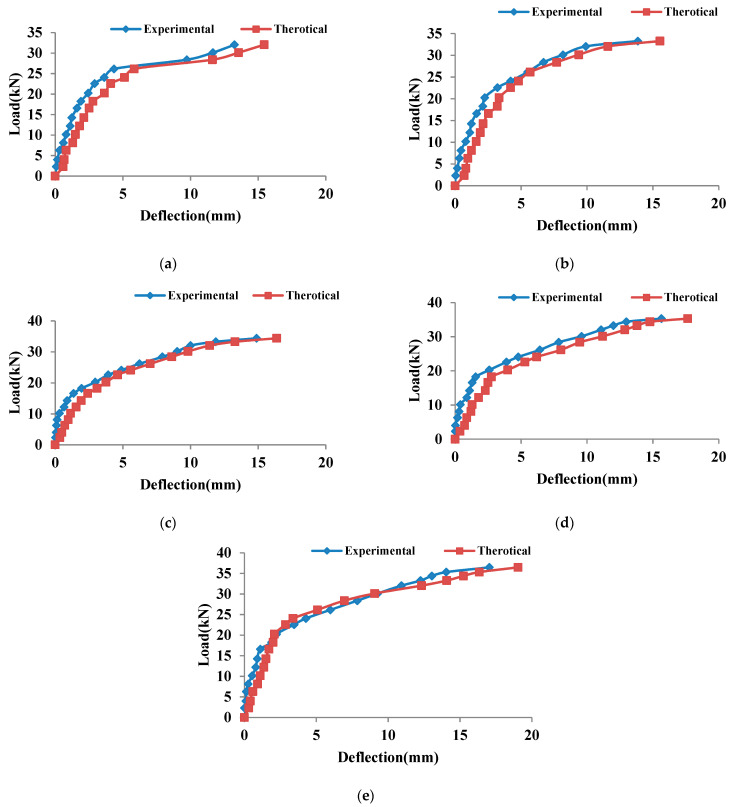
The comparison of the experimental and predicted deflection of (**a**) CS, (**b**) CSF25, (**c**) CSF50, (**d**) CSF50, and (**e**) CSF100.

The theoretical deflection of all of the beams was computed and compared to the experimental deflection using the suggested modified Branson’s equation, as illustrated in Figure 10a–e.

#### 3.2.5. Ductility

The ductility of the reinforced concrete members was determined by calculating the ductility ratio. A higher ductility ratio shows that a beam may withstand significant deflections before failing [72]. There are several techniques to define ductility, which can be classified into two broad categories: energy and deformation-based methods [24]. The most frequently used technique is one based on deformation, as indicated by the deformation margin between the ultimate stage and service stage [64,72]. The ductility ratio was calculated using the deformation technique, as shown in Equation (6).

The flexural ductility of a structure is determined by comparing it to a reference condition obtained from the internal reinforcement yield point. The structural component may sustain load, despite undergoing significant deformations due to the distinctive yield plateau of the stress–strain plot [73]. The ductility ratio is used to determine the ductility of reinforced concrete members. A greater ductility ratio indicates that a structural member may withstand considerable deflections without failing [30]. The deformation technique, as illustrated in Equation (6), was used to compute the ductility ratio.

μ_D_ = ∆_u_/∆_y_ (6)

where μ_D_ represents the deformation-based ductility ratio; ∆_u_ represents the ultimate mid-span deflection; and ∆y represents the mid-span deflection during the service stage.

The comparison of the different ductility ratios of plain and fiber-reinforced coconut shell beams is shown in Table 5. The CS beam’s ductility ratio is around 4.9, which is close to previous studies [30]. Because of its low modulus of elasticity, coconut shell aggregate is a ductile material. The low modulus of elasticity of coconut shell concrete allows for significant deformation of the concrete component, resulting in high strain for increased load [30]. It is clear from this experiment that adding steel fiber lowered the deformation-based ductility of steel fiber-reinforced coconut shell concrete beams, and increasing the proportion of fiber further reduced the ductility.

It can be shown that the CSF25 beam had a 12% drop in ductility, while the CSF100 mix had a ductility value of 65% of the CS beam. Members with a ductility ratio of 3 to 5 have acceptable ductility, according to Ashour [74], and can be regarded as structural members exposed to significant deformation such as aseismic stresses. The ductility ratio of all fiber-reinforced beam specimens in this investigation was between 3.2 and 4.9. All other specimens had adequate ductility values, indicating that they meet the structural stability criterion.

**Figure 11:**
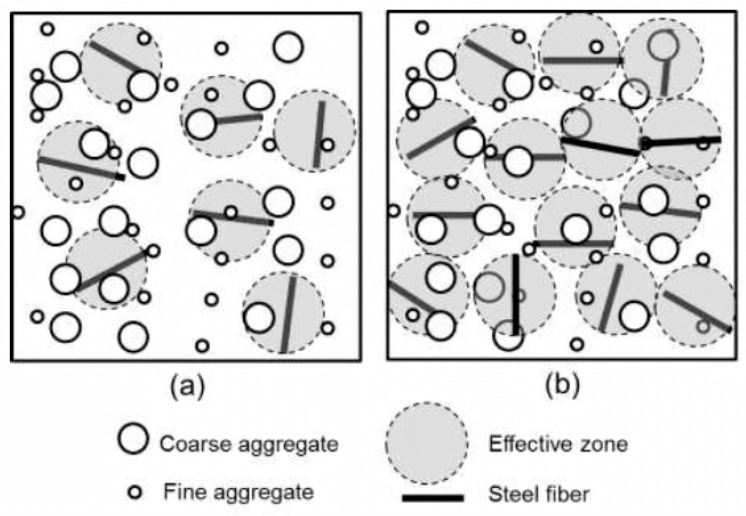
The effect of confinement by steel fibers for (**a**) low fraction and (**b**) high fraction.

The ductility ratio D was calculated solely on the basis of the deformation of the steel fiber-reinforced coconut shell concrete beams and did not take into account the enhancing effect of steel fibers on the load carrying capacity. As a result, as the volume of fibers increased, the ductility ratios decreased. According to Gao et al. and Okay and Engin [75,76], a minimum amount of steel fiber (0.3–1.5%) is necessary to improve the technical characteristics and structural behavior of concrete. In addition, the minimum volume fraction of steel fibers that must be added to significantly increase the mechanical characteristics and moment capacity is 0.50%. Figure 11 depicts the confinement effect of fibers. It was observed that when the steel fiber addition was less than 0.5%, the impact of confinement was not as strong as when the steel fiber addition was greater than 0.50% [77]. The effective stress zone of the steel fibers was not linked to one another when the volume of the fiber fraction is low (less than 0.5%), and thus the impact of confinement due to the steel fibers is weak. The steel fiber-reinforced coconut shell concrete specimens’ load bearing capacity improved due to the strong confinement effect. The confinement effect causes strain localization, resulting in early steel-reinforcement fracture [63].

Flexural toughness is the measure of energy absorption. The toughness of the material was determined by finding the area under the load–deflection plot. The flexural toughness values of all of the specimens are shown in Table 6. The toughness results revealed that the CSF25, CSF50, CSF75, and CSF100 beams showed a 10–45% increase in the toughness over the control beam CS. Steel fibers, for example, boost the toughness of both the pre-peak and post-peak performance in steel fiber-reinforced coconut shell concrete. The flexural toughness of a material is a measure of its energy absorption capacity. Table 6 shows the flexural toughness values of all of the specimens. The toughness results showed that the CSF25, CSF50, CSF75, and CSF100 beams were 10–45% tougher than the control beam CS. For instance, steel fiber-reinforcement improved the toughness of both the pre-peak and post-peak performance in steel fiber-reinforced coconut shell concrete beams.

### 3.3. Numerical Validation

ANSYS was used to simulate beams with sizes of 100 mm × 150 mm × 1500 mm using real material characteristics, with an increasing number of smaller components. The reaction parameters of beams are the bottommost fiber’s deflection at mid span and the topmost fiber’s compressive stress at mid span. If the model has a significant number of smaller parts, the results will converge. Convergence issues may occur as the mesh density increases. Only trial solutions are used to determine the needed mesh density. To create a stiff model, all of the nodes were fused together. A total of five beams were modeled for bending. Control beams were also modeled and compared with other fiber reinforced mixes. The size and cover provided to the beams were kept constant at 100 × 150 × 1500 mm and 20 mm, respectively, for all beams. For beams, two 10 mm diameter bars at the bottom and two 8 mm diameter bars at the top were given. Shear reinforcement was given by two-legged stirrups with a 6 mm diameter at 100 mm *c*/*c*.

The following element types were used in the simulation by ANSYS Workbench:

- SOLID 65, BEAM188, TARGET170

SOLID65 is used to simulate solids with or without reinforcing bars in three dimensions (rebar). The solid can rupture under strain and crush under compression. The element is defined by eight nodes, each of which has three translational degrees of freedom in the *x*, *y*, and *z* axes. There can be up to three distinct rebar specifications defined.

The consideration of nonlinear material properties is the most critical part of this element. The concrete can crack (in three orthogonal directions), crush, distort plastically, and creep. The reinforcing bar can withstand tension and compression, but not shear. Additionally, they can undergo plastic deformation and creep. BEAM188 is appropriate for assessing thin to moderately stout/thick beam structures. The element is founded on the Timoshenko beam theory, which accounts for the shear–deformation phenomena. The element gives options for unconstrained and constrained cross-section warping.

By default, the element incorporates stress stiffness terms in any study with significant deflection. The provided stress–stiffness terms allow for elements to examine the flexural, lateral, and torsional stability issues (using eigenvalue buckling, or collapse studies with arc length methods or nonlinear stabilization).

TARGE170 is utilized for a variety of 3D ‘target’ surfaces and the accompanying contact elements such as CONTA173, CONTA174, CONTA175, CONTA176, and CONTA177. The contact elements themselves overlay the solid, line elements, or shell, so characterizing the border of a deformable body, and may be in contact with the target surface, as described by TARGE170.

#### 3.3.1. Material Properties

Creating a model for concrete behavior is a difficult endeavor. Concrete is quasi-brittle and behaves differently under compression and strain. In this investigation, material nonlinearity was utilized. Table 7 displays the anticipated nonlinear material properties for concrete.

From the experimental results, the elastic modulus and flexural strength of all the combinations were determined. The FEA comprised the modeling of beams with experimentally-determined sizes and mechanical properties. To generate the FEM in ANSYS, numerous steps must be undertaken for the model to function correctly. Models may be produced using either a graphical user interface or a command prompt.

#### 3.3.2. Finite Element Discretization

In the first step of the FEA, the beam model is meshed. The beam model is subdivided into numerous smaller pieces during meshing. After applying a load, the stress and strain are determined at the points of integration of smaller parts. Mesh density selection is one of the primary processes in finite element modeling. When a model contains a sufficient number of elements, the convergence of outcomes is achieved. Practically, this is achieved when an increase in mesh density has a negligible impact on the outcomes. In order to determine the optimal mesh density for this finite element modeling, a convergence analysis was conducted. The finite element models duplicated the dimensions of the full-scale beams. With an increasing number of smaller parts, 100 mm × 150 mm × 1700 mm beams with their real material properties were modeled using ANSYS. The response parameters for beams are the deflection of the bottommost fiber at midspan and the compressive stress on the topmost fiber at midspan. The convergence of findings is attained when a sufficient number of smaller model elements are utilized. When the density of the mesh is increased, convergence issues may emerge. The needed mesh density is determined solely from trial solutions. All of the nodes were blended together to create a rigid model.

#### 3.3.3. Meshing

**Figure 12:**
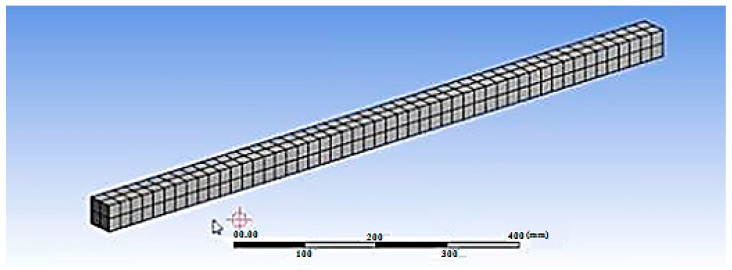
The meshed model.

To obtain accurate results from the SOLID65 element, a higher order 3D solid element with 20 nodes and quadratic displacement behavior is employed. With the aid of the meshing tool menu, meshing is performed. The mesh tool menu contains a global set holding the size of the element’s divisions, which determines the element’s size. As the number of elements increases as the size of the element lowers, the findings obtained are accurate. As the number of elements increases, so does the time required to solve the problem under the given load, necessitating an increase in computer memory capacity. Due to the extremely small diameter of reinforcing bars, the above-described process for meshing reinforcing bars necessitates a significant reduction in the bar element size. The meshed model is shown in Figure 12.

#### 3.3.4. Loads and Boundary Conditions

The finite element models were loaded in the identical places as the actual beams. The model must be constrained by displacement boundaries in order to yield a unique output. To ensure that the model behaves identically to the experimental beam, boundary conditions are implemented at the points of symmetry where the supports and loads exist. The beam was designed with a hinged support at one end and a roller at the opposite end.

#### 3.3.5. Analysis Methodology for FE Model

**Figure 13:**
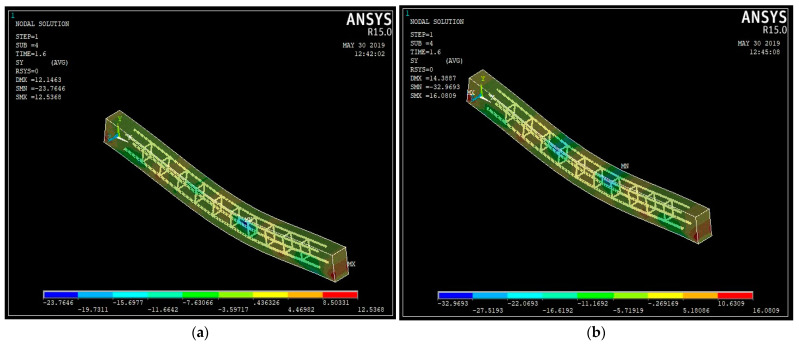

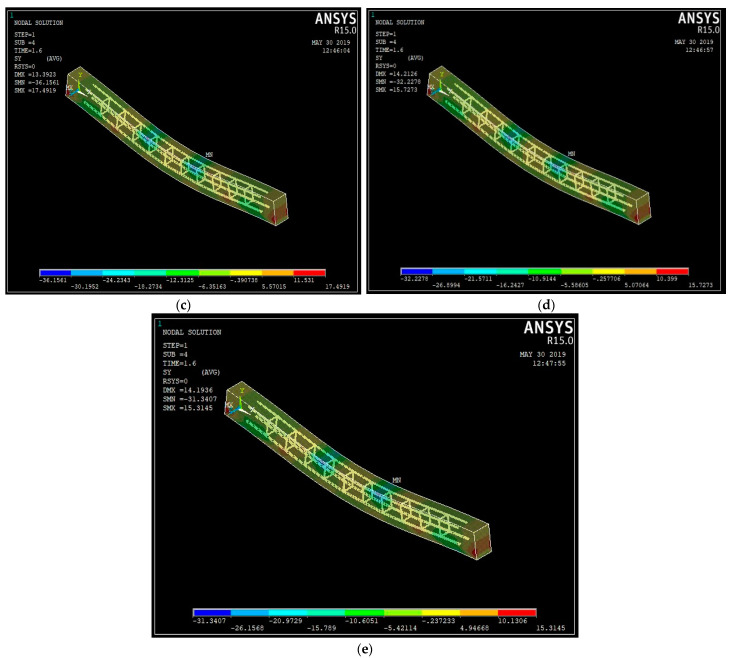
The stress contour of the steel fiber-reinforced coconut shell concrete beams: (**a**) CS; (**b**) CSF25; (**c**) CSF50; (**d**) CSF75; (**e**) CSF100.

**Figure 14:**
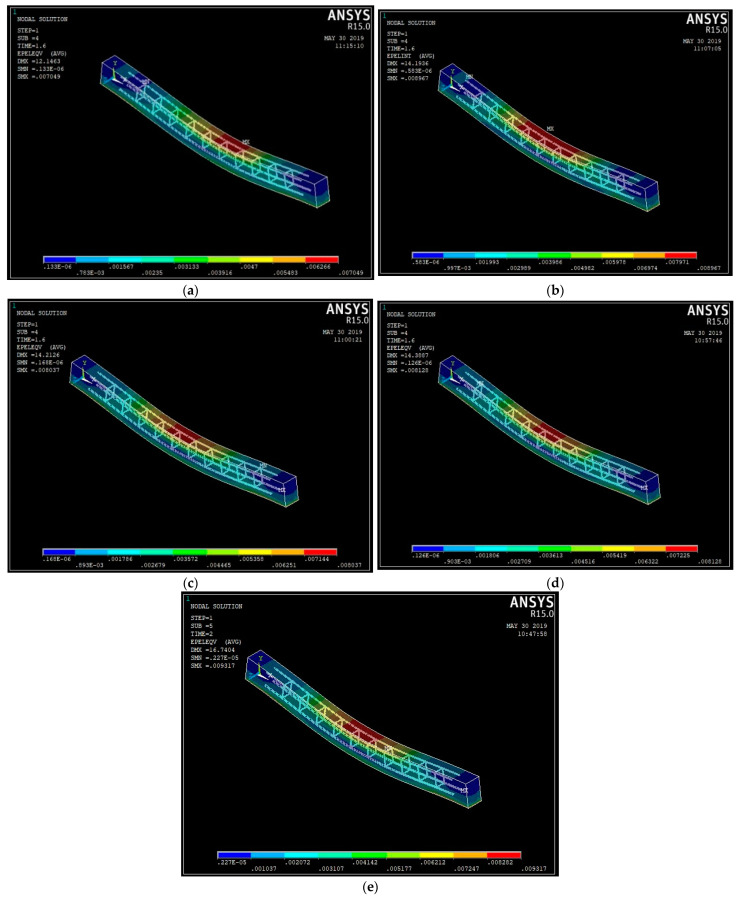
The strain plot of the steel fiber-reinforced coconut shell concrete beams: (**a**) CS; (**b**) CSF25; (**c**) CSF50; (**d**) CSF75; (**e**) CSF100.

The model’s FEA was configured to investigate the beam’s various behaviors. Linear analysis was performed to identify the deflection, stress–strain graphs, and experimental values for validation. Newton–Rapshon equilibrium iterations are used in ANSYS software. Newton–Rapshon equilibrium iterations give convergence within tolerance limits at the conclusion of each load phase. To avoid the divergence issue, a force convergence criterion with a tolerance limit of 5% was applied. The number of equilibrium iterations required was increased to 100. Table 8 summarizes the finite element model results and the experimental tests for the beams. The results suggest that the ANSYS numerical analysis is capable of accurately predicting the failure load and deflection up to service load. Figure 13a–e illustrates the stress contours for the beams with varying % ages of steel fibers. Figure 14a–e shows the strain graphs for the ultimate loads on the steel fiber-reinforced coconut shell concrete beams.

The stress contour shown in Figure 12 indicated that the maximum stress was observed in the regions where the actual cracks were formed on the experimental observations. As the load increased, the steel reinforcement was found to deform substantially but the beam failed due to the formation of flexural cracks in the concrete.

On observing the strain pattern obtained from the finite element analysis from Figure 13, it can be noted that the red color contour indicates that it is the zone of formation of the plastic hinge and they are the points where actual failure had taken place. The strain pattern observed from the finite element analysis exactly matched that of the failure pattern obtained from the experimental investigation.

## 4. Conclusions

This research explored the outcomes of the experimental and theoretical analyses of the steel fiber-reinforced sustainable concrete made with coconut shell, an agricultural waste, as coarse aggregate and class F fly ash, an industrial by-product as a partial substitute for cement. The influence of steel fiber at up to 1% volume fraction on the compressive strength and flexural characteristics of fiber-reinforced coconut shell concrete were investigated. The experimental flexural characteristics were compared with the analytical as well as numerical study developed in this investigation. The following conclusions were drawn.

- Steel fiber inclusion enhanced the compressive strength of coconut shell concrete from 15% to 39%.
- A typical flexural failure was seen in plain coconut shell concrete beams and steel fiber-reinforced coconut shell concrete beams.
- Adding steel fibers to coconut shell concrete beams enhanced their ultimate moment capacity by 5–14%. When the steel fiber content was more than 0.5%, the moment capacity and crack resistance of the steel fiber-reinforced coconut shell concrete beam specimens were enhanced significantly.
- It was found that when compared to the non-fibrous beams, all fiber-reinforced beams showed a reduction in ductility, which can be attributed to strain localization. The ductility ratio for structural ductility was met by all of the fiber-reinforced coconut shell concrete beams.
- The experimental moment capacity of all of the beams was found to be higher than the theoretical moment capacity. Because the equations specified in codes such as IS and BS are not intended for fiber-reinforced concrete components, the observed moment capacity and displacements of the steel fiber-reinforced coconut shell concrete beams either overestimated or underestimated the theoretical values.
- The steel fiber addition increased the flexural toughness of the coconut shell concrete beams up to 45%.
- According to IS456 and BS 8110, the span/deflection ratio of all of the fiber-reinforced coconut shell concrete beams was found within the permitted range.
- For the fiber-reinforced coconut shell concrete beam, Branson’s model was developed, and the findings were in good agreement with the experimental data.
- The finite element models were created and analyzed using ANSYS. The findings of all of the finite element beams demonstrated that the ultimate loads were close to the experimental values. The measured deflection agreed reasonably with the deflection derived from the finite element calculations.

Coconut shell concrete with the partial replacement of fly ash can be used as an eco-friendly building material because coconut shell is a renewable and naturally available resource while fly ash is a type of industrial waste. The addition of steel fiber further enhanced the mechanical and flexural performance of coconut shell concrete, thereby making it feasible for structural applications. Consequently, the development of steel fiber-reinforced coconut shell concrete with fly ash blends will contribute to the sustainability of the concrete industry.

## Figures and Tables

**Table 1:** The properties of the coconut shell aggregate.

Physical and Mechanical Characteristics	Values
Size (mm)	12.5–4.75
Thickness (mm)	3–7
Moisture content (%)	4.4
Moisture absorption (%)	24
Specific gravity	1.10
Abrasion value (%)	1.9
Impact value (%)	8.0
Crushing value (%)	2.3
Bulk Density (kg/m^3^)	650

**Table 2:** The mix proportions.

Mix ID	Fly Ash (kg/m^3^)	Cement (kg/m^3)^	Sand (kg/m^3^)	CS agg. (kg/m^3^)	Conv. agg. (kg/m^3^)	*w*/*b*	SP (%)	Steel Fiber (%)
CS	51	459	750	332	0	0.33	1.2	0
CSF25	51	459	750	332	0	0.33	1.2	0.25
CSF50	51	459	750	332	0	0.33	1.2	0.5
CSF75	51	459	750	332	0	0.33	1.2	0.75
CSF100	51	459	750	332	0	0.33	1.2	1.0

Note: CS agg.—Coconut Shell aggregate; Conv. agg.—Conventional aggregate; SP—Superplasticizer.

**Table 3:** The ultimate moment.

Beam ID	Experimental Value		Theoretical Value		Capacity Ratio Exp./Theor.
	Load (kN)	Moment (kNm)	Load (kN)	Moment (kNm)	
CS	32.074	8.09	31.13	7.78	1.04
CSF25	33.532	8.47	31.36	7.84	1.08
CSF50	34.4	8.71	31.45	7.87	1.11
CSF75	35.32	8.93	31.65	7.91	1.13
CSF100	36.42	9.26	31.72	7.93	1.17

**Table 4:** The deflection of the beams.

Beam ID	Midspan Deflection at Service Load (mm)	IS 456:2000 and BS 8110 (Span/250) Upper Limit Permissible Deflection, (mm)	Δ exp/Δ per	Span/Δ exp	Midspan Deflection at Ultimate Load
CS	2.7	6.0	0.45	555	13.28
CSF25	3.2		0.53	469	13.88
CSF50	4.0		0.67	375	14.9
CSF75	4.5		0.75	333	15.67
CSF100	5.4		0.9	278	17.04

**Table 5:** The ductility indices.

Beam ID	Service Stage		Ultimate Stage		Disp. Ductility Ratio μ_D_ = ∆_u_/∆_y_
	Load (kN)	Disp. (∆_y_) (mm)	Load (kN)	Disp. (∆_u_) (mm)	
CS	21.35	2.7	32.074	13.28	4.9
CSF25	22.17	3.2	33.532	13.88	4.3
CSF50	22.93	4.0	34.4	14.90	3.7
CSF75	23.55	4.5	35.32	15.67	3.5
CSF100	24.31	5.4	36.42	17.04	3.2

**Table 6:** The flexural toughness.

Beam ID	Flexural Toughness kN·mm	Percentage Variation over Control Specimen
CS	328.99	-
CSF25	362.70	10.25
CSF50	394.84	20.02
CSF75	420.29	27.75
CSF100	476.13	44.72

**Table 7:** The material properties.

Material	Properties	Values
Concrete	Poisson’s ratio	0.18
	Grade of concrete	M35 (based on trial mix)
	Modulus of elasticity	according to fiber volume added
	Modulus of rupture	The fiber volume added
	Shear transfer coefficient for open crack	0.2
	Shear transfer coefficient for closed crack	1
Steel	Poisson’ ratio	0.3
	Grade of steel	Fe 415
	Young’s Modulus	2.0 × 10^5^ MPa
	Yield strength	415 MPa
	Tangent modulus	41.5 MPa

**Table 8:** The comparison of the ANSYS results with the experimental results.

Beam ID	Comp. Strength at 28 Days	Failure Load (kN)			Total Deformation (mm)		
		Expt.	ANSYS	A/E	Expt.	ANSYS	A/E
CS	35.6	32.07	31.02	0.967	13.28	12.14	0.914
CSF25	40.8	33.53	34.13	1.018	13.88	14.19	1.022
CSF50	43.1	34.4	34.50	1.003	14.90	14.21	0.954
CSF75	47.8	35.32	35.25	0.998	15.67	14.39	0.918
CSF100	49.5	36.42	36.50	1.002	17.04	16.49	0.968

## Data Availability

Data sharing not applicable.
